# Facile lipase-catalyzed synthesis of a chocolate fat mimetic

**DOI:** 10.1038/s41598-018-33600-x

**Published:** 2018-10-15

**Authors:** Saeed M. Ghazani, Alejandro G. Marangoni

**Affiliations:** Department of Food Science, University of Guelph, Guelph, Ontario, Canada

## Abstract

A cocoa butter equivalent (CBE) was synthesized enzymatically from readily available edible fats with fatty acid and triacylglycerol compositions that closely resemble the fat present in chocolate, cocoa butter. A commercially available immobilized fungal lipase, Lipozyme RM IM, was used as the reaction catalyst. Reaction parameters were a temperature of 65 °C, water activity of 0.11, a 4 h reaction time, and a substrate mass ratio of a commercial enzymatically synthesized shea stearin (SS) to palm mid-fraction (PMF) of 6:4 (w/w). Fractionation was also used after reaction completion to further approach the triacylglycerol composition of cocoa butter by removing trisaturated and unsaturated triacylglycerols. The yield of the triglyceride 1-palmitoyl-2-oleoyl, 3-stearoyl-glycerol (POS) produced was 57.7% (w/w). The amounts of 1,3-dipalmitoyl-2-oleoyl-glycerol (POP), (POS) and 1,3-stearoyl-2-oleoyl-glycerol (SOS) in the final CBE were 11.2%, 36.3%, and 34.8%, respectively. In comparison, the amounts of POP, POS and SOS in the cocoa butter used in this study were 15.2%, 38.2%, and 27.8%, respectively. No significant differences (*P* > 0.05) in melting point and enthalpy of fusion between CB and the CBE were observed. In comparison, a non-interesterified blend of SS and PMF (60:40 w/w) showed significantly (*P* < 0.05) higher melting point and lower enthalpy of fusion compared to CB. The crystal polymorphic form V of CB (β_2-_3L) was similar to that of CBE and SS/PMF (60:40 w/w). The solid fat content (SFC) vs. temperature profile of the CBE generally resembled that of CB, except that the CBE had significantly (*P* < 0.05) higher SFCs at 5, 10, 15, 20 and 25 °C compared to both CB and SS/PMF (60:40 w/w). Addition of 15% (w/w) CBE to CB did not cause any changes in physical properties (melting point, SFC and crystal polymorphic forms) of the CB. This study demonstrates the potential for synthesizing a CB-like CBE using a green, rapid, straightforward one step enzymatic conversion followed by fractionation from widely available edible fats.

## Introduction

Cocoa butter (CB) is one of the most expensive fats in the world and current trends in the cocoa butter market suggest further rises in cocoa butter prices, shortages, strong demand in emerging countries, and profit squeezes for companies in the future. This means that finding a practical alternative is necessary. Over the years, many attempts have been made to produce cocoa butter alternatives using different methods such as blending of cheaper and readily available oil and fat sources and their derivatives, chemical interesterification (intra-, trans- or directed methods), hydrogenation (selective or non-selective), and fractionation (dry, detergents or solvents)^1^. Various natural tropical seed fats contain one or two of the three main triacylglycerols (TAG) present in cocoa butter (CB), POP, POS and SOS, but none contain the three TAGs at levels equivalent to CB^2^.

Cocoa butter alternatives are categorized as CB equivalents (CBEs), CB replacements (CBRs), and CB substitutes (CBSs). Among these groups, CBRs and CBSs are incompatible with cocoa butter due to the marked differences in TAG molecular composition. CBR and CBS generally contain lauric (high in 12-carbon saturated fatty acids) or *trans* fats, which have a very different molecular composition than CB. Thus, blending CBRs and CBSs with CB can lead to changes in the physical properties of the final product such as decreases in the melting point, solid fat content and hardness, as well as changes in the crystal polymorphic form. This rarely enhances the sensory quality of chocolate and instead results in defects such as waxiness, softening and loss of gloss, and accelerated bloom development^2^.

Moreover, due to the removal of partially hydrogenated oils (PHOs) from the Generally Recognized as Safe (GRAS) list has caused a drastic reduction in CBR use in the confectionary industry. In contrast, CBS alternatives originating from palm kernel oil or coconut oil contain high amounts of lauric acid in their TAG profile. These provide a sharp melting point similar to CB, and can crystallize directly from the melt into a stable polymorphic form without tempering, but cannot be mixed freely with CB due to eutectic softening effects^3^. On the other hand, CBEs are the most compatible CB alternatives. The first CBE produced at an industrial scale (Coberine) was made by merely blending equal parts of palm mid-fraction (PMF) and illipe butter by Loders Croklaan^1^. The main natural sources for CBEs are exotic butters such as sal fat, shea butter, kokum butter, mango kernel fat, and illipe butter^4^. The supply of exotic butters is very limited since they are extracted from the seeds of wild trees growing in tropical forests. Moreover, quality can also be very variable from year to year^5^. So, partial or full replacements of CB with these exotic butters have limitations. These natural CBEs also lack one or two of the main structural TAGs of cocoa butter (POP, POS and SOS). For example, Chinese vegetable tallow and pequi oil, only have high amount of POP, while sal, shea, kokum, mango kernel fats are only rich in SOS. The only exotic butter rich in both SOS and POS is illipe butter^6,7^.

Enzymatic interesterification (EIE) of fats and oils to produce specialty fats has gained considerable attention since regio-specific microbial lipases (3.1.1.3) offer certain advantages over chemical catalysts^8^. These include reaction products with a TAG composition that more closely resembles cocoa butter (1,3- specific reaction instead of random), the possibility of running the reaction at lower temperatures, enzyme re-use, lower isomerization by-products, and better control of CBE production^9^. Currently, microbial lipases from *Rhizomucor*, *Rhizopus*, and *Candida* such as Lipozyme TL IM from *Mucor miehei* and Novozyme 435 from *Candida antarctica* are commercially available^10^.

Developments in lipase immobilization techniques on novel supports such as ion-exchange resins, microporous polypropylene, silk fibers, and nanofibers drastically reduced enzymatic modification costs and removed economic barriers related to the industrial scale application of lipase in the modification of fats and oils^10,11^.

Early research on CBE production using EIE methods was mainly based on conducting the reaction in organic solvent media^12–15^. This had some benefits, such as minimizing hydrolysis, increased yield^16^, decreased viscosity of the reaction medium^17^, simplified recovery of products, by-products and lipase at the end of the reaction^18^, and maintaining the catalytic activity of lipases at higher temperatures^19^. Currently, most industrial scale EIE reactions for CBE synthesis are conducted in solvent free systems because of safety and environmental concerns, using high substrate concentrations in the absence of solvents^20^. Bloomer *et al*.^14^ reported that EIE in solvent free systems had the advantage of a decreased trisaturated TAG formation. For the first time in the 1970s, Loders Croklaan used immobilized *Mucor miehei* lipase in a packed-bed reactor to synthesize a CBE by the acidolysis reaction between palm mid-fraction (PMF) and stearic acid. Later, the company switched from using PMF to high oleic sunflower oil^21^. Around the same period of time, Fuji Oil used different base stocks (blend of fatty acid esters and TAGs) to produce CBEs^1^.

CBE synthesis using EIE can be categorized into two main groups: acidolysis and interesterification reactions. Acidolysis reactions are the most popular method of fats and oils modification for the confectionary industry. An example of an acidolysis reaction of high oleic canola oil (OOO) with palmitic (P) and stearic (S) acids is shown in Equation 1.

1 $$OOO+S+P\to POP+POS+SOS+POO+SOO+OOO+P+S+O+MAGs+DAGs$$

At the end of the reaction, the residual palmitic and/or stearic free fatty acids are usually stripped using molecular distillation, followed by post-fractionation in order to decrease the amount of tri-saturated TAGs and DAGs in the hard fraction, and OOO, POO and SOO from soft fraction and obtaining a CBE as the mid-fraction^11,22^. Mohamed^23^ prepared a CBE through EIE of PMF with palmitic and stearic acids and the composition of the CBE at the end of the reaction was: POP 30.7%, POS 40.1%, POO 9.0%, SOS 14.5%, and SOO 5.7%, with a melting point of 40.4 °C. The main concerns about industrial scale acidolysis reactions include the high cost of the distillation process at the end of the reaction to remove free stearic and palmitic acids and the low yield of reaction products because of the presence of high amounts of DAGs and tri-saturated TAGs^24–26^.

In a recently published paper, a CBE was produced through EIE of high oleic sunflower oil (HOSO) or high oleic/high stearic sunflower oil (HOHS) as the main source of POO, SOO, and OOO with a mixture of stearic and palmitic acid (oil/fatty acids molar ratios were 1:7 and 1:5 respectively). The reaction was run at 65 °C for 8 hours^27^. The product yields from acidolysis with HOSO and HOHS were determined to be 36% and 54% respectively. The total amount of POP, POS, and SOS in CBEs synthesized with HOSO and HOHS were 74.6% and 78.0% respectively. The effect of EIE of HOSO with different acyl donors, including stearic acid, methyl stearate, and vinyl stearate, using three commercial lipases (Lipozyme RM IM, Lipozyme TL IM, and NS40086) on the yield of SOS was studied by Wang *et al*.^28^. They showed the highest SOS yield (24% to 50.6%) was obtained when stearic acid was used as the acyl donor. Moreover, increasing the molar ratios of HOSO to stearic acid from 1:6 to 1:12 caused the yield of the reaction to increase to 70%. Gibon^11^ showed that in an optimized enzymatic acidolysis reaction in a batch system, after a 32-hour reaction between PMF and stearic acid (50/50% w/w), the amounts of POP, POS, and SOS were 22%, 30%, and 10%, respectively. The total amounts of tri-saturated TAGs (PPP, PPS, PSS, and SSS), on the other hand, were 10%. Response Surface Methodology (RSM) was applied to optimize enzymatic acidolysis catalysed by Lipozyme RM IM between HOSO and a mixture of stearic acid and palmitic acid. At the end of the reaction, a low yield of desired TAGs (POP, POS, and SOS) was obtained and the total yield after distillation and fractionation steps was 15.4%. In addition, about 65% stearic and palmitic acids remained in the reaction media^24^. A mixture of PMF and stearic acid was selected as the base stock to synthesize CBE by using Lipozyme RM IM at 65 °C by Undurraga *et al*. After distillation, the amount of POP, POS, SOS, and DAGs were 23.4%, 38.5%, 20.2%, and 9.7% respectively. The yield of the reaction was between 80–90%. The existence of some high melting peaks in the CBE melting thermogram was attributed to the presence of DAGs and trisaturated TAGs (PPS and PSS) after distillation^25^. Mohammed^23^ reported the synthesis of a CBE by EIE of PMF and a mixture of palmitic and stearic acids (1:2 molar ratio) using a batch reactor at 60 °C. The reaction resulted in the production of a CBE with the TAG composition: POP 30.7%, POS 40.1%, POO 9.0%, SOS 14.5%, and SOO 5.7%. The melting temperature of the CBE before distillation was 40.4 °C, i.e. much higher than that of form V of CB. In another study, refined olive pomace oil and a mixture of palmitic and stearic acids (1:2:6 molar ratio) was utilized to synthesis a CBE using Lipozyme RM IM as the catalyst. The TAG composition of the final CBE was: POP 11%, POS 21.8%, and SOS 15.7%, and its melting point was 29.9 °C. That is also outside of the acceptable melting range for CB (32–35 °C)^29^.

The second group of EIE reactions for CBE production is based on the reaction between a fat or an oil (mostly high oleic canola oil or high oleic sunflower oil) and palmitic and/or stearic acid ethyl or methyl esters (Equation 2).

2 $$\begin{array}{rcll}OOO+Ethyl \mbox{-} S+Ethyl \mbox{-} P & \to  & POP+POS+SOS+POO+SOO & \,\,+\,Ethyl \mbox{-} O+Ethyl \mbox{-} S+Ethyl \mbox{-} P\end{array}$$

This method was used on an industrial scale for the first time by the Fuji Oil Company for CBE production based on EIE of ethyl stearate and PMF. Ethyl stearate as the acyl donor compared to stearic acid has the benefits of lower melting point, boiling point, and higher vapour pressure. Therefore, at a specific temperature, the viscosity of the synthesized products would be lower and a lower distillation temperature can be applied for purification^1^. The main disadvantages of using ethyl esters are their higher costs and the unavoidable partial hydrolysis during EIE and increasing FFA content. Moreover, recovery of glycerol esters (TAGs and DAGs) from by-products is more complicated^22^.

Early research on transesterification between exotic butters and methyl esters was conducted by Sridhar *et al*.^30^. The goal of that study was to increase the amount of POS in synthesized CBEs. Selected Indian fats (mango kernel, mahua, kokum, sal, and dhupa butters) and methyl palmitate and stearate were selected as substrates to run EIE. In a downstream step, the products were passed through a silica gel column using ethanol (95%) as the carrier solvent to separate the by-products from the final product. The final separated components were: TAGs (63%), methyl esters (21.4%), and DAGs (10.5%). The highest yield of obtained pure TAGs was 70%. Comparing the results among different exotic butters, TAG composition of EIE kokum butter was quite similar to CB (POP 16%, POS 37%, and SOS 29%). The melting point of the EIE kokum butter was decreased from 41.2 °C to 32.8 °C. Later in 1995, Gitlesen *et al*.^31^ compared the effects of the stearic acid donor (methyl stearate or stearic acid) on the yield of EIE reaction products. They reported that, when high oleic rapeseed oil reacted with methyl stearate, the resulting distribution of the main acylglycerols was: SOO 27%, SOS 36%, and DAGs 6%. When high oleic rapeseed oil was reacted with stearic acid instead, the distribution was 30% SOO, 20% SOS and 20% DAGs. Moreover, acyl migration (relocation of stearic acid to the s*n*-2 position) was significantly lower when stearic acid than with methyl stearate. In a study in 2006, Wang *et al*.^32^, ran EIE reactions between tea seed oil and a methyl palmitate and methyl stearate mixture (1:8:8 molar ratio) using pancreatic lipase as the catalyst at 35 °C for 60 hours. Although the CBE produced had a similar melting point to CB (33.3 °C vs 37.7 °C), the yield of the reaction based on the weight of the substrate was only 25.6%.

The transesterification reaction between two different fats (usually PMF as one of the base stocks) is the last group of CBE synthesis methods by EIE. For instance, in Equation 3, PMF is interesterified with shea stearin and in Equation 4 fully hydrogenated canola oil is reacted with PMF.

3 $$POP+SOS\to POP+POS+SOS+MAGs+DAGs$$

4 $$POP+SSS\to POP+POS+SOS+PPS+PSS+SSS+MAGs+DAGs$$

High amounts of trisaturated TAGs at the end of the reaction are one of the main disadvantages of using fully hydrogenated oils. Moreover, running the reaction at high temperatures (higher than the melting point of fully hydrogenated oils) causes acyl migration and formation of undesirable TAG structures. So running this type of EIE reaction in solvent is a necessity. In 1990, Chang *et al*.^33^, synthesized a cocoa butter-like fat through EIE of a fully hydrogenated cottonseed oil and olive oil mixture. The maximum POS content was produced after 4 hours on and the highest yield of the reaction was 19% w/w. The melting point of the final product after a two-step solvent fractionation was 39 °C. In a similar study^34^, the CBE yield produced through EIE of PMF and tristearin using Lipozyme RM IM in supercritical carbon dioxide was 53%. A similar base stock (blend of palm oil and fully hydrogenated soybean oil) was used by Abigor *et al*.^35^ to produce a CBE using EIE. This was followed by acetone fractionation and silica chromatography to remove MAGs, DAGs, and high melting TAGs. The DSC melting thermogram of the final CBE showed a “shoulder” around 38 °C that could be related to the presence of residual tri-saturated TAGs.

The main purpose of this study was to design and optimize a lipase-catalyzed enzymatic reaction to synthesize a cocoa butter equivalent using readily available commercial stocks. In recent work from our lab, we identified and characterized a “shea stearin” like fat from microalgae^36^. This fat is referred to as “algal butter” and showed very similar physico-chemical characteristics to commercial shea stearin, but originates from microalgae grown in reactors. Thus, algal butter is not subject to natural availability; it can be grown at will at large scales. Even though the work presented here was carried out on commercial enzymatically synthesized shea stearin, our work has shown that the molecular composition of commercial shea stearin and algal butter are very similar, thus results from this study can be applied on algal butters, once their supply increases. We believe that the strategy of using transesterification of two fats is the most practical and promising in consideration of availability of materials, cost, ease of production, downstream processing, and sustainability. The main target of this work was obtaining the highest amount of POS and the lowest amount of tri-saturated TAGs in the shortest reaction time. At the end of the reaction, high melting TAGs (StStSt) and low melting TAGs (StOO) will be removed using solvent fractionation. However, dry fractionation could achieve the same goal, but modern industrial-scale fractionation units would be required. Solvent fractionation is used here only as proof of principle.

## Materials and Methods

### Materials

Commercial refined, bleached, and deodorized (RBD) enzymatically synthesized shea stearin (SS) used in this study was received as a gift from Fuji Oil Co., LTD. (Fuji Oil, Osaka, Japan). A commercial RBD palm mid-fraction (PMF) sample was received as a gift from AAK Co., LTD. (AAK Denmark A/S, Aarhus C, Denmark) and Malaysian RBD cocoa butter was supplied from JB Cocoa Sdn. Bhd. (JB Cocoa, Johor, Malaysia). A granular 1,3 specific Lipozyme^®^ RM IM, (*Rhizomucor miehei* lipase) from Novozyme Company) that was immobilized on a macroporous anion-exchange resin (Duolite ES 562) with a molecular weight of 31,600 Da, was a gift from Bunge Oils, Inc., (Bradley, IL, USA). Molecular sieves, 4 Å were purchased from Sigma-Aldrich Company (Sigma-Aldrich Canada Co., Oakville, Canada). Molecular sieves were dried at 100 °C for 16 hours before addition to the reaction medium.

### Fatty acid composition determination

An Agilent 6890-series gas chromatograph (Agilent Technologies, Inc., Wilmington, DE, USA) with a 7683-series auto-sampler was used to define the FA composition of the samples. A 60 m × 0.22 mm internal diameter with a 0.25 μm film thickness GC column BPX70 (SGE Inc. Austin, TX, USA) was used. The oven temperature was programmed to increase from 110 to 230 °C (4 °C/min) and was maintained at 230 °C for 18 minutes. The injector temperature was set at 250 °C and operating at 20.1 psi with a flow of 17.7 mL/min. Helium, a carrier gas, flowed at an average velocity of 25 cm/s. A flame ionization detector was set at 255 °C with 450 mL/min air and 50 mL/min helium flowing. The GC separation peaks were analyzed using Open LAB software (Agilent Technologies). Fatty acid composition was determined by comparing retention times of the peaks to internal standards.

### Triacylglycerol composition determination

The TAG composition of the samples was determined using a Waters Alliance model 2690 high performance liquid chromatography with a refractive index detector (Waters model 2410, Waters, Milford, MA, USA). Chromatographic separation of the DAGs and TAGs in the samples was obtained with a Waters xbridge C18 (Waters Limited, Mississauga, ON, Canada) column (4.6 mm × 250 mm internal diameter with 5 μm particle size). Isocratic elution with a flow rate of 1 mL/min of degassed acetone/acetonitrile 60/40 (v/v) was applied. Column and detector temperatures were set at 40 °C. The data obtained was analyzed using Millenium32 (K&K Testing, LLC, Decatur, GA, USA). The TAG composition was determined using internal standards and their corresponding retention time.

### Water activity (*a*_*w*_) measurement

The water activity of the samples was analysed using an AquaLab water activity meter (Model 4TE DUO, Decagon devices, USA). The mechanism of measurement is based on determination of the dew point temperature of air equilibrated with the sample. In addition, an infrared thermometer indicates the sample temperature. Three replicates were applied for each measurement. To remove excess moisture from the immobilized lipase, the enzyme was first reacted with the substrate for 30 minutes (drying lipase) at 65 °C, followed by a filtration step. Then, molecular sieves were added (15% w/w) to absorb water in the reaction medium. The molecular sieves (zeolite) used in this study were crystalline sodium aluminosilicate with a diameter of 2.4–4.8 mm and 4 Å pore diameter, widely used for adsorption of water (critical diameter of 3.2 Å). Since molecular sieves have a microporous structure, they entrap or adsorb different types of molecules based on their size and the strength of adsorption interactions that take place on the internal pore surface and on the particle surface. They have high affinity to separate various components from gases (hydrogen, oxygen, nitrogen and carbon dioxide) to linear and branched hydrocarbons. Moreover, some EIE reaction by-products such as free fatty acids and glycerol as polar components have affinity to adsorb onto molecular sieves; however, it depends on their molecular size^37^.

### Enzymatic interesterification reaction

EIE reactions were carried out in 250-mL Erlenmeyer flasks with phenolic screw caps in a solvent free system using a water bath shaker to mix the reaction media while maintaining constant reaction temperature. After preparation of a base stock mixture (60% w/w SS-40% w/w PMF blend), immobilized lipase (10% w/w of substrate) and molecular sieves (15% w/w of substrate) were added to the substrate and mixed. Pre-conditioning step was applied to eliminate air and reducing the amount of moisture in lipase with the following procedures; first entrapped air in immobilized lipase granules and in molecular sieves was removed using a vacuum pump (50 mm/Hg). Then, substrate was esterified with de-aired lipase for an hour at 65 °C and at the end, the reaction stopped and esterified oil was filtered and lipase was collected. The second step was repeated for three times to reduce the moisture content of lipase as low as possible. After pre-conditioning step, lipase was added to the substrate, the EIE reaction was started at 65 °C^35^. Samples were taken from the reaction flasks at specific time intervals (0–19 hours) and filtered to remove immobilized enzymes and molecular sieves and stored at −18 °C until analysis. Even though in this study the reaction was carried out in a batch system, and at the end of the reaction the molecular sieves and lipase were filtered, it is possible to carry out the reaction in a continuous system and using packed molecular sieves in a column to remove water. Previous studies have shown that during an EIE reaction under optimum conditions, Lipozyme RM IM retained over 80% of its original activity, even after 16 passes through a continuous bed reactor^38^.

### Solvent fractionation

Acetone fractionation was performed to remove high melting and low melting TAGs from the cocoa butter-like TAGs (POP, POS, and SOS). The ratio of fat:acetone was 1:4 w/w for all fractionations. EIE fat crystallized from acetone at two different temperatures. The first crystallization was conducted at 22 °C to remove mostly tri-saturated TAGs in a solid phase and a second fractionation was conducted to remove low melting TAGs at 14 °C and produce the CBE.

### Melting point and enthalpy of fusion

Melting points and enthalpies of fusion for the samples were obtained using a differential scanning calorimeter (DSC) model Q2000 (TA instruments, Mississauga, ON, Canada). Nitrogen was used as the purge gas with the flow of 18 mL/min. In this experiment, melting points (endothermic peak) of samples (2–5 mg) were determined by heating from 5 °C to 60 °C at the heating rate of 5 °C/min.

### Crystal structure and polymorphism

The crystal structure and polymorphic form of fats were analysed by X-ray diffraction (Multiflex Powder XRD spectrometer, Rigaku, Tokyo, Japan). The copper X-ray tube (wavelength of 1.54 Å) was operated at 40 kV and 44 mA. The measurement scan rate was set at 0.1°/minute in the range 2θ = 1°−30° at 20 °C. Peak positions were determined using MDI Jade 9 (MDI, Livermore, CA, USA) software.

### Solid fat content (SFC)

SFC was determined according to the AOCS Official Method Cd 16–81 by pulsed Nuclear Magnetic Resonance (NMR) Spectrometer (Bruker mq20 Minispec Series PC 120, Milton, ON, Canada) operating at 20 MHz and 0.47 T. The MiniSpec software V2.51 Rev 00/NT (Bruker Biospin Ltd., Milton, ON, Canada) was used to analyse data.

### Microstructure by polarized light microscopy (PLM)

The microstructure was imaged by PLM using an optical microscope model BX60 (Olympus Optical Co., Tokyo, Japan) equipped with a 20× objective lens. Images were captured (20×) with a model DP71 digital camera (Olympus Optical Co., Tokyo, Japan) using the v1.0 cellSens software. A 5 μL sample of each molten fat was placed on a preheated (80 °C) glass microscope slide and covered with a preheated (80 °C) glass coverslip to remove excess fat and air pockets. Slides were heated at 80 °C for 15 minutes to erase the crystal memory, then cooled to 22 °C and held for one week prior to observations.

### Statistical Analysis

Statistical analysis was carried out using GraphPad Prism software version 5.0 (La Jolla, CA, USA). All analyses were run in duplicates and results were stated as mean values ± standard deviations. Data were evaluated using one‐way ANOVA and probability of *p* < 0.05 was considered as significant.

## Results and Discussion

### Optimizing the reaction

#### Water activity (*a*_*w*_)

**Figure 1:**
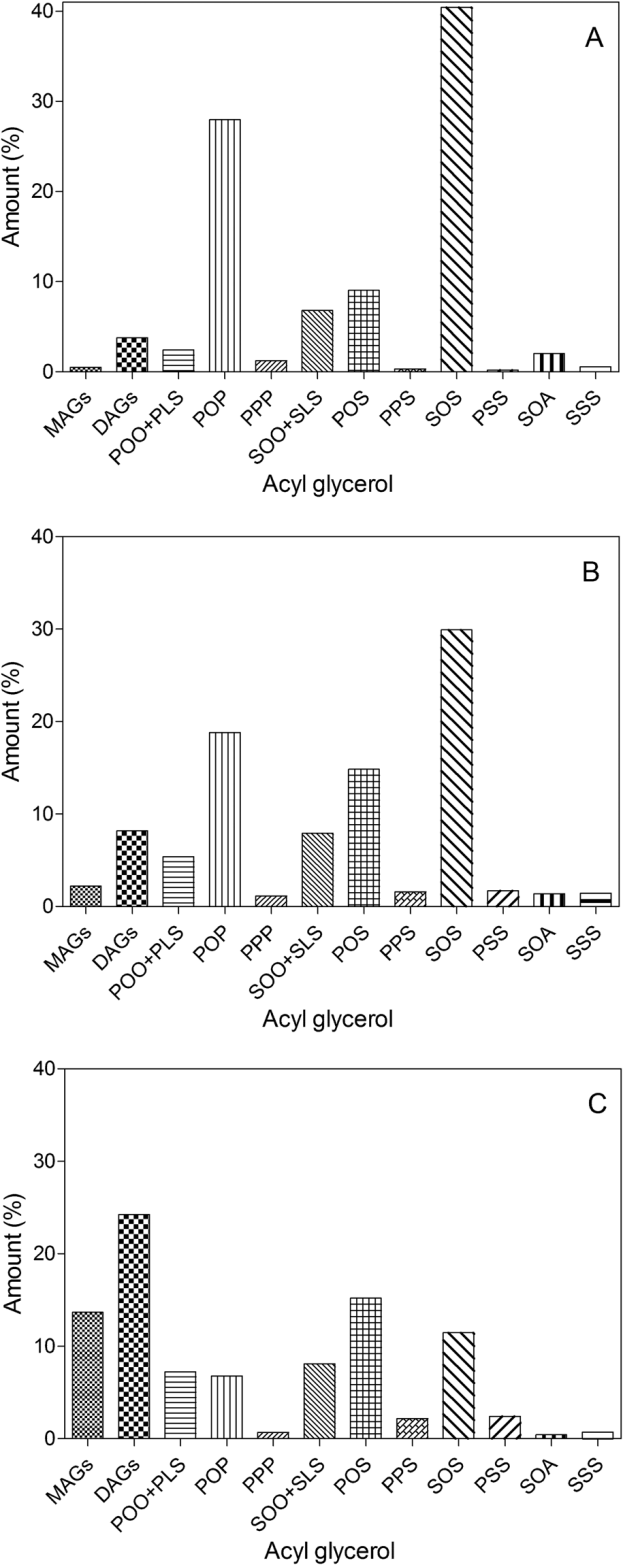
The effect of pre-treatment step on the formation of different acylglycerols (MAGs, DAGs and TAGs) during the enzymatic interesterification reaction.

Removing water before starting the reaction and controlling the moisture content during EIE is one of the main factors that affects final product yield and formation of partial acylglycerols (MAGs and DAGs) and free fatty acids. In the presence of excess water, the equilibrium of the reaction shifts in favor of hydrolysis. Limiting the water content of the reaction shifts the equilibrium towards the interesterification reaction products. Thus, continuously removing water from the reaction media is necessary^39^. However, a certain amount of water is also necessary to maintain the activity of the immobilized lipase^40^. Moreover, the reaction rate and yield of EIE are influenced by the water content of the system^41^. Yamane^42^ showed that at a lower moisture levels, the reaction yield was higher, while the rate of the reaction was lower. Several methods can be applied for water removal from the reaction mixture, such as applying a vacuum, bubbling dry air or N_2_ through the oil, and/or using drying agents such as molecular sieves, silica gel, and azeotropic solvent distillation^20,43,44^. Previous research has shown that the addition of molecular sieves to an EIE reaction has the main advantage of controlling the water activity of the system^45^. This reduces the extent of hydrolysis reactions and formation of by-products (mainly free fatty acids and DAGs)^46^ and increasing the reaction yield by shifting the thermodynamic equilibrium towards ester synthesis^47^. The water activity of Lipozyme RM IM (in powder form) before starting the reaction was 0.423 ± 0.004 at 25 °C. The water activity of Lipozyme RM IM in oil (added at a 10% w/w level) was decreased from 0.212 ± 0.007 to 0.112 ± 0.001 as a result of the drying and addition of molecular sieves. Pinyaphong and Phutrakul^26^ showed the best performance for the EIE of palm oil and methyl stearate using *Carica papaya* lipase as the catalyst was obtained at *a*_*w*_ = 0.11. The effects of the drying method on acylglycerol composition of EIE fat (SS + PMF 60:40) are shown in Fig. 1. It is clear from Fig. 1 that the presence of moisture in immobilized lipase caused a dramatic increase in the amounts of MAGs and DAGs formed. These amounts before starting the reaction were around 0.5% and 3% respectively. The amounts of MAGs and DAGs in the EIE sample catalyzed with dried lipase increased to 2% and 8% respectively. EIE with non-dried lipase caused the MAGs and DAGs content to increase to 14% and 24%, respectively. Drying steps did not have a significant effect on POS yield at the end of the reaction (15% for both treatments), although it was effective in increasing the amount of SOS and POP in the final CBE (SOS 30% and POP 19% with dried lipase compared to SOS 11.5% and POP 7% with non-dried lipase). Owusu-Ansah,^1^ showed that molecular sieves and vacuum were effective at removing water from the EIE reaction. In this study, before starting the reaction, a vacuum (50 mm/Hg) was applied to remove entrapped air from the immobilized lipase and molecular sieves. One of the greatest effects of moisture in EIE reactions is on acyl migration. In an EIE reaction, higher acyl migration was obtained with increasing moisture content^48^. However, Kadivar *et al*.^49^ showed that water did not induce acyl migration in EIE in solvent free systems. Lipozyme RM IM immobilized on a macroporous resin was less influenced by the moisture content. Additionally, it can be reduced to a very low amount during the EIE reaction^50^. Kim *et al*.^51^ compared the effects of three water amounts (300, 650, and 1000 mg/kg) during EIE of high oleic sunflower oil and an ethyl palmitate and stearate mixture on total symmetrical TAGs, POS content, and acyl migration. No significant effect on these parameters was detected, but water level significantly and positively influenced the DAG content. In this study, a combination of vacuum and molecular sieves was used to remove water from the reaction.

### Temperature

Temperature is another critical factor affecting CBE production. It greatly influences reaction rate, product yield, acyl migration, and the amount of trisaturated TAGs, and DAGs at the end of reaction^51–53^. When synthesizing CBEs through EIE, the reaction temperature should be high enough (>60 °C) to keep the substrates in the liquid form (fatty acids, methyl- or ethyl ester of fatty acids, or TAGs). However, very high temperatures can reduce the reaction rate due to denaturation of the lipase^21^. The optimum reaction temperature for Lipozyme RM IM is between 60–70 °C^49^. A study showed that during synthesis of a CBE using Lipozyme RM IM, acyl migration started at 70 °C, while no TAG positional isomers were formed at 60 °C^49^. Kim^51^ showed that increasing the reaction temperature from 40 °C to 60 °C had no effect on POS content, while it significantly and positively affected acyl migration and had a negative effect on DAG levels. In their study, the optimized temperature of 60 °C was selected through response surface methodology. In our study, a reaction temperature of 65 °C was thus chosen for the EIE reaction.

### Substrate ratio

Mimicking the fatty acid composition of CB was the initial step to design a proper CBE with the highest compatibility with CB. For this purpose, PMF and SS (sources of palmitic acid and stearic acid, respectively) were selected as substrates for the reaction. Palm oil fractions are readily available at a low cost in the market compared to exotic butters. In this study, we used a commercial enzymatically synthesized shea stearin, but a novel shea stearin from algal sources has been reported with similar physicochemical properties to the shea stearin used in this study^54^. The fatty acid compositions of PMF and SS, as commercial CBEs, were studied by Padley^55^. The fatty acid profile for PMF was reported as palmitic acid 56%, stearic acid 6%, oleic acid 32%, and linoleic acid 4% and for SS the fatty acid profile was palmitic acid 5%, stearic acid 57%, oleic acid 33%, and linoleic acid 3%. The fatty acid profiles for commercial PMF and SS used in this study were quite similar to these results, as shown in Table 1. Based on the amounts of palmitic acid and stearic acid in PMF and SS (57.4% and 56.4% respectively), a SS/PMF (60:40 w/w) blend was selected as the base stock for the EIE reaction. Fatty acid composition of the SS/PMF (60:40 w/w) blend is shown in Table 1. No significant differences (*P* > 0.05) between the fatty acid composition (C16:0, C18:0, C18:1, C18:2, and C20:0) of the blend and CB were determined. Since the amount of POP, POS, and SOS in natural CBEs (palm oil and exotic butters) are not high enough to match those present in CB, a fractionation technique (dry or solvent fractionation) is usually applied to increase the concentration of these TAGs to mimic CB^55^. CBEs are usually produced by blending PMF and one or more high SOS sources. In most cases it is not possible to match the POS content in CB by this strategy. Similarly, our PMF/SS (60:40 w/w) blend had low levels of POS, with TAG compositions of 28.0%, 9.1%, and 40.5% POP, POS, and SOS, respectively.

**Table 1:** Kinetic parameters obtained for enzymatic interesterification of SS/PMF (60:40 w/w) blend.

	K	[C_0_]	[C_max_]	[C_plateau_]	[C_span_]	R^2^
POP	1.283 ± 0.046	26.77 ± 0.17	—	11.16 ± 0.11	15.61 ± 0.20	0.9986
POS	1.472 ± 0.066	9.53 ± 0.26	29.24 ± 0.15	—	—	0.9980
SOS	1.560 ± 0.236	41.70 ± 0.80	—	23.11 ± 0.47	18.59 ± 0.92	0.9789
StOO	0.786 ± 0.061	9.35 ± 0.18	17.04 ± 0.16	—	—	0.9977
StStSt	0.369 ± 0.039	2.25 ± 0.11	8.53 ± 0.30	—	—	0.9981
MAGs	0.109 ± 0.023	0.51 ± 0.14	—	2.60 ± 0.11	2.09 ± 0.17	0.9870
DAGs	0.117 ± 0.001	3.78 ± 0.01	—	8.66 ± 0.01	4.88 ± 0.01	1.0000

### Reaction progress and kinetic model

CBE yield and reaction by-products are greatly influenced by the reaction time. Even though CBE yield increases in time, so do reaction by-products (MAGs and DAGs) and undesirable TAGs^56^.

**Figure 2:**
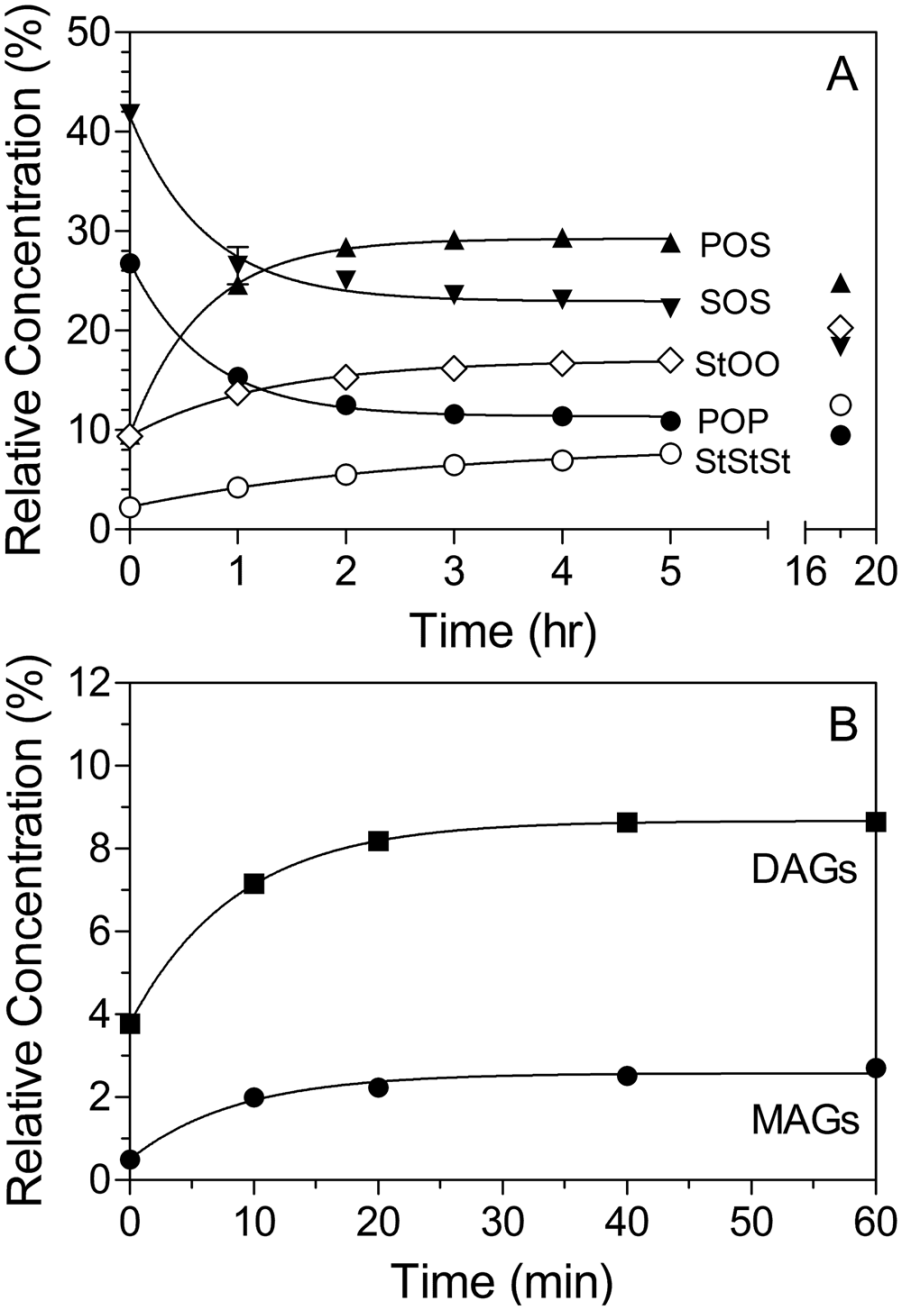
Lipase-catalyzed enzymatic interesterification reaction progress as function of time. The EIE reaction was performed at 65 °C using Lipozyme RM IM (10% w/w) as catalyst.

We thus monitored changes in TAGs, DAGs and MAGs time, which are shown in Fig. 2. In order to quantify the kinetics of this reaction, enzymatic transesterification between POP and SOS was modelled according to the following kinetic scheme. The first step in a lipase-mediated transesterification is the formation of a diglyceride and a lipase-fatty acid acyl complex. The fatty acyl complex is between the active site serine and a fatty acid which was esterified onto positions *sn-1* or *sn*-3 on the TAG molecule. This process will take place for both SOS and POP, and can be depicted as follows:

5 $$SOS\to SO+Enz-S$$

6 $$POP\to PO+Enz-P$$

where PO and SO correspond to 1-palmitoyl, 2-oleyl diglyceride and 1-stearoyl, 2-oleyl diglyceride, respectively, while Enz-S and Enz-P correspond to the stearic and palmitic acid acyl complexes with the enzyme, respectively.

The second step of the reaction, assuming no acyl migration, would involve the reaction of the Enzyme-fatty acid complex with the diglycerides, namely

7 $$SO\mathop{\longrightarrow }\limits^{Enz-P}SOP,\,\,SO\mathop{\longrightarrow }\limits^{Enz-S}SOS,\,PO\mathop{\longrightarrow }\limits^{Enz-S}POS,\,PO\mathop{\longrightarrow }\limits^{Enz-P}POP$$

All these reactions are actually reversible, however, we are stating here that the reaction is effectively irreversible, in order to simplify the kinetic scheme. This reversibility and the fact that the reaction does plateau and approach equilibrium will be taken into account within the kinetic model by including maxima and minima in the functions (see below). Here we also assume that the rate limiting step of the reaction is the formation of diacylglycerols, SO and PO. Once these are formed, the enzyme fatty acyl complex rapidly reacts with the diglycerides to form the desired product POS (=SOP). Thus, we will model the reaction using a simple first order decay model, with limits, for both SOS and POP degradation and a simple first order association model, with limits, for POS formation.

The final functions for this model are as follows:

8 $$POP(t)=PO{P}_{\min }+(PO{P}_{0}-PO{P}_{\min }){e}^{-kt}$$

9 $$SOS(t)=SO{S}_{\min }+(SO{S}_{0}-SO{S}_{\min }){e}^{-kt}$$

10 $$POS(t)=PO{S}_{o}+(SO{S}_{0}-SO{S}_{\min }+PO{P}_{0}-PO{P}_{\min })\cdot (1-{e}^{-kt})$$

The fits of the model to the data are shown in Fig. 2. All Pearson r^2^ values were greater than 0.97 and, in most cases, greater than 0.99. Moreover, the numerical value of “k”, the rate constant for hydrolysis and esterification was statistically the same for the three reactions (*P* > 0.05), and hence stated as such in the kinetic model above. SOO + POO formation, PPP + PPS + SSP (=StStSt, or tri-saturated TAG) formation and monoglyceride (MAG) and diglyceride (DAG) accumulations were modeled using similar functions. Enzymatic interesterification kinetic parameters are shown in Table 1. Based on the results, the initial POS concentration before starting the reaction was 9.1%. The maximum POS content (29.3%) was obtained after 4 hours of reaction, while after only 1 hour, approximately 79% progress in POS production had been achieved. Furthermore, as shown in Fig. 2, the concentration of POS decreased beyond 4 hours. Meanwhile after 4 hours of reaction, the SOS and POP contents had decreased and reached a plateau at 23.1% and 11.2%, respectively. Therefore, the interesterification reaction reached near-equilibrium in 4 hours. Macrae^57^ showed a rapid EIE rate can be obtain if the interfacial area between the substrate and lipase is large. Applying a vacuum degassing step to remove air entrapped in granules of immobilized lipase before starting the reaction substantially increased this surface area.

The total amounts of SOO and POO (StOO) and PPP, PPS, PSS, and SSS (StStSt) increased from 9.4% to 17.0% and from 2.3% to 8.5% respectively after 4 hours (Table 1). After an 18 hour reaction period, the amount of StOO and StStSt increased to 20.3% and 12.5% respectively. Furthermore, the total amounts of MAGs and DAGs increased and reached a plateau at 40 minutes (MAGs, 2.6% and DAGs, 8.7%). Throughout the EIE reaction, DAGs are formed as reaction intermediates and are considered as the originator of EIE side reactions (migration of acyl groups from *sn*-1,3 to *sn*-2) through acyl migration^58^.

The base stocks of the reaction, PMF and SS, contained around 4% and 3% DAGs respectively. The main DAGs in palm oil and its derivatives are PP, PO, and OO^59^. During EIE, acyl migration occurred from the *sn-*1,3 positions to the *sn-*2 position, increasing the amount of saturated fatty acids in the *sn*-2 position of the TAGs (higher PPS, PSS, and SSS contents). Acyl migration of oleic acid from the *sn*-2 position to the *sn*-1,3 positions in DAGs led to increases in the concentration of POO and SOO as by-products of the EIE reaction. Since high melting TAGs (StStSt) and low melting TAGs (StOO) cause a drastic change in the physical and functional properties of the final CBE, acyl migration needs to be minimized^14^. DAGs play a key role in the initiation of acyl migration while other reaction parameters such as higher enzyme load, moisture, temperature, and longer reaction time also affect acyl migration^59^. Bloomer *et al*.^14^ showed that a high amount of trisaturated TAGs at the end of an acidolysis reaction (triolein and palmitic acid) was a result of spontaneous acyl migration of MAG and DAG intermediates. In their study, during the first 40 minutes of the reaction, the concentration of DAGs increased rapidly and then showed a slow reduction at a constant rate. Moreover, no PPP was formed in the first hour of the EIE reaction. The authors concluded that a high enzyme load (10%), the lowest reaction temperature (60 °C), and the use of dried Lipozyme resulted in the elimination of PPP and reduction of DAGs from reaction products. In the enzymatic acidolysis of sunflower oil with a palmitic and stearic acid mixture, reaction time and temperature positively influenced acyl migration^60^. In contrast, lower enzyme load resulted in less acyl migration^51^. In a CBE produced using lard and tristearin as the substrate in supercritical carbon dioxide, a three-hour reaction was optimal to achieve the highest POS content^61^.

The total amount of major TAGs (POP, POS and SOS) in our CBE after four hours of EIE reaction was 63.8%, while this amount in solvent fractionated CBE, CB and SS:PMF (6:4 w/w) blend was 82.3%, 81.8% and 62.3%, respectively. The ratio of POS to (POP + POS + SOS) for our CBE after four hours of EIE reaction was 46%, while for CB and SS + PMF (60:40 w/w) was 41.4% and 11.7%, respectively (Table 3).

### Fatty acid and Triacylglycerol Composition

Natural non-lauric fats and exotic butters mostly contain a simple mixture of saturated and mono-unsaturated fatty acids (palmitic, stearic, and oleic acids) which account for over 90% of those present. In tropical plants that produce exotic butters in their fruits or seeds, various metabolic pathways are employed to synthesize TAGs consisting mainly of POP, POS, and SOS and POO and SOO in lower amounts. More than 38 different types of natural butters from various plant species having high amounts of palmitic, stearic, and oleic acids have been identified. These include cocoa butter, phulwara butter, shea butter, baku butter, dumori butter, njatuo tallow, kokum butter, bacury butter, lamy butter, allanblackia fat, illipe butter, borneo tallow, sal butter, malabar tallow, laurel fat, khakan fat, and mango butter^62^.

The fat extracted from cocoa seeds (*Theobroma cacao*), contains mainly palmitic (24.9–27.1%), stearic (32.9–37.3%), and oleic (33.1–37.6%) acids comprise over 95% of the fatty acids present. There is also a low amount of linoleic acid present (2.3–3.7%)^62^. Cocoa beans originated in Latin America and from there they spread throughout the world. Today, cocoa butter can be obtained from Ivory Coast, Ghana, Indonesia, Cameroon, Nigeria, Brazil, Ecuador, Dominican Republic, and Malaysia^63^. The molecular species composition of the different CBs depends on their origin. Asian CBs (Malaysian and Indonesian) contain less palmitic and more stearic acid than South American CBs (Brazilian), which contain a higher amount of oleic acid^64^. Fatty acid compositions of CBs are shown in Table 2. The fatty acid composition of the Malaysian CB used in this study was in the range reported for Malaysian CB by Jahurul *et al*.^63^ (palmitic 24.9–26.0%, stearic 36.0–37.4%, oleic 33.5–34% and linoleic acid 2.6–3.0%).

**Table 2:** Fatty acid composition (% w/w) of PMF, SS, blend SS/PMF (60:40 w/w), CB and CBE.

	C16:0	C18:0	C18:1	C18:2	C20:0
SS	5.33 ± 0.23^a^	56.39 ± 0.28^a^	33.50 ± 0.30^a^	2.93 ± 0.04^a^	1.71 ± 0.01^a^
PMF	57.41 ± 0.13^b^	6.22 ± 0.00^b^	31.65 ± 0.03^b^	3.31 ± 0.15^b^	nd
CBE	25.37 ± 0.18^c^	42.48 ± 0.12^c^	30.15 ± 0.44^c^	0.19 ± 0.01^c^	1.30 ± 0.00b
CB	25.96 ± 0.09^d^	37.10 ± 0.24^d^	32.49 ± 0.18^b^	2.88 ± 0.00^a^	1.21 ± 0.01c
SS/PMF (60:40%)	26.85 ± 0.12^e^	35.81 ± 0.05^e^	32.63 ± 0.01^a,b^	3.03 ± 0.01^a^	1.19 ± 0.00c

Different superscript letters within each column indicate significant differences (*P* < 0.05) among samples.

The TAG compositions of the blend as well as the CBE before and after solvent fractionation are compared with CB in Table 3. During EIE, MAGs and DAGs are produced as intermediate reaction products^65^. Obtaining the lowest amounts of MAGs and DAGs at the end of the enzymatic reaction was one of the main targets in this study. The main DAGs in CB are 1,3-PO 25%, 1,3-SO 25%, and 1,3-PS 24%. The total amount of DAGs in CB is typically 1.1–2.8%^65^. The different effects of DAGs on fat crystallization have been studied by many researchers, such as lowering the crystallization rate^66^, slowing down the kinetics of crystallization, both in terms of nucleation and crystal growth^67,68^, acting as crystal seeds and increasing the onset of crystallization^69^, or delaying the polymorphic transition of TAGs^27^. No significant difference (*P* < 0.05) in the total amount of MAGs in the CB, CBE, and the blend was detected. The total amount of MAGs in the CBE before and after solvent fractionation did not change significantly (from 1.1% to 0.8%), while the total amount of DAGs increased from 5.1% to 8.1%. The total amount of DAGs in the CB and the blend were 2.3% and 3.8% respectively (Table 3). The TAG composition of CB is dominated by three main TAGs (POP, POS, and SOS) that account for over 80% of those present. The ratio between TAGs in CB can also be a way to locate the geographical origin of CB. For instance, POO + PLS contents can be plotted against SOO + SLS contents^70^. South American CBs contain higher amounts of SOO and SLS compared to Asian CBs, which have a high level of POS and SOS^64^. Our blended fat contained a higher amount of POP and SOS and low levels of POS compared to CB. So, mimicking the TAG composition of CB by blending these stocks is not possible even though the fatty acid composition of the blend was quite similar to that of CB. A true mimetic needs to have similar TAG molecular species as CB, not usually reflected in the fatty acid composition of cocoa butter. A modification technique (more than just blending) is thus necessary to modify the TAG composition of the blend. The POP, POS, and SOS contents in the CB used in this study were 15.2%, 38.9%, and 27.7% respectively. The amount of these TAGs in Ivory Coast CB reported by Hartel *et al*.^71^ were 14.5%, 38.6%, and 28.4% respectively.

**Table 3:** Triacylglycerol composition (% w/w) of CB, CBE before and after fractionation, CB/CBE (85:15 w/w), SS, PMF and SS/PMF (60:40 w/w).

TAG	CB	CBE after 4 hrs EIE	CBE after fractionation	SS/PMF 60/40%	CB/CBE 85/15%	SS	PMF
POO + PLS	5.15 ± 0.25^a^	7.53 ± 0.03^b^	1.11 ± 0.16^c^	2.42 ± 0.05^d^	5.13 ± 0.03^a^	1.66 ± 0.01^c,e^	3.64 ± 0.23^e^
POP	15.22 ± 0.13^a^	11.38 ± 0.20^b^	11.17 ± 0.12^b^	27.97 ± 0.46^c^	15.77 ± 0.25^a^	2.66 ± 0.14^d^	61.52 ± 0.75^e^
PPP	0.39 ± 0.18^a^	1.08 ± 0.03^a^	1.26 ± 0.03^a^	1.24 ± 0.01^a^	0.33 ± 0.04^a^	nd	3.87 ± 0.75^b^
SOO + SLS	4.50 ± 0.01^a^	9.16 ± 0.00^b^	1.39 ± 0.12^c^	6.81 ± 0.04^d^	3.83 ± 0.25 ^e^	10.95 ± 0.07 ^f^	nd
POS	38.88 ± 0.53^a^	29.34 ± 0.25^b^	36.32 ± 0.04^c^	9.06 ± 0.22^d^	39.76 ± 0.79^a^	5.96 ± 0.23^e^	12.50 ± 0.06 ^f^
PPS	0.52 ± 0.07^a^	2.74 ± 0.03^b^	1.75 ± 0.01^c^	0.34 ± 0.06^a^	0.49 ± 0.01^a^	nd	0.82 ± 0.16^d^
SOS	27.73 ± 0.06^a^	23.09 ± 0.12^b^	34.77 ± 0.41^c^	40.45 ± 0.50^d^	28.72 ± 0.71^a^	66.52 ± 0.43^e^	1.43 ± 0.03 ^f^
PSS	0.59 ± 0.11^a^	3.12 ± 0.02^b^	0.63 ± 0.01^a^	0.20 ± 0.00^c^	0.38 ± 0.03^a^	0.31 ± 0.01^c^	nd
SOA	1.59 ± 0.08^a^	0.60 ± 0.25^b^	1.82 ± 0.11^a^	2.04 ± 0.08^c^	1.42 ± 0.00^a^	3.50 ± 0.08^d^	nd
SSS	0.32 ± 0.04^a^	1.42 ± 0.08^b^	0.16 ± 0.01^a^	1.10 ± 0.01^b^	0.23 ± 0.04^a^	2.18 ± 0.37^c^	nd
MAGs	0.96 ± 0.16^a^	1.09 ± 0.01^a^	0.83 ± 0.01^a^	0.49 ± 0.27^b^	1.47 ± 0.10^c^	nd	0.49 ± 0.04^b^
DAGs	2.27 ± 0.00^a^	5.13 ± 0.16^b^	8.06 ± 0.08^c^	3.75 ± 0.91^b,d^	3.32 ± 0.87^a^	3.05 ± 0.21^d^	3.89 ± 0.16^b,d^
POP + POS + SOS	81.83	63.81	82.26	77.48	84.25	75.14	62.34
POS/ POP + POS + SOS	41.4	46.0	44.2	11.7	47.2	7.9	20.1
Total StStSt	1.82	8.36	3.80	2.88	1.43	2.49	4.69

Different superscript letters between each column indicate significant differences (*P* < 0.05) among samples.

### Solvent fractionation

Solvent fractionation (using acetone, acetonitrile, or hexane) is one of the most common industrial scale processes to separate high value-added TAG products at the end of EIE reactions. The main advantages of solvent fractionation compared to dry or detergent fractionation are improved separation efficiency and higher fractionation yield^56^. Solvent fractionation was an effective method to improve the TAG composition of the CBE to more closely resemble that of CB. The POS and SOS concentrations increased significantly (*P* < 0.05) from 29.3% and 23.1% respectively, before fractionation, to 36.3% and 34.8% after solvent fractionation. Moreover, the total amount of saturated TAGs (StStSt) was decreased from 8.4% before fractionation to 3.8% after fractionation (55% reduction). Additionally, the total amount of di-unsaturated TAGs (StOO) was reduced from 16.7% before fractionation to 2.5% after fractionation (85% reduction). However, solvent fractionation increased the content of DAGs from 5% to 8%.

### Ternary phase diagram

**Figure 3:**
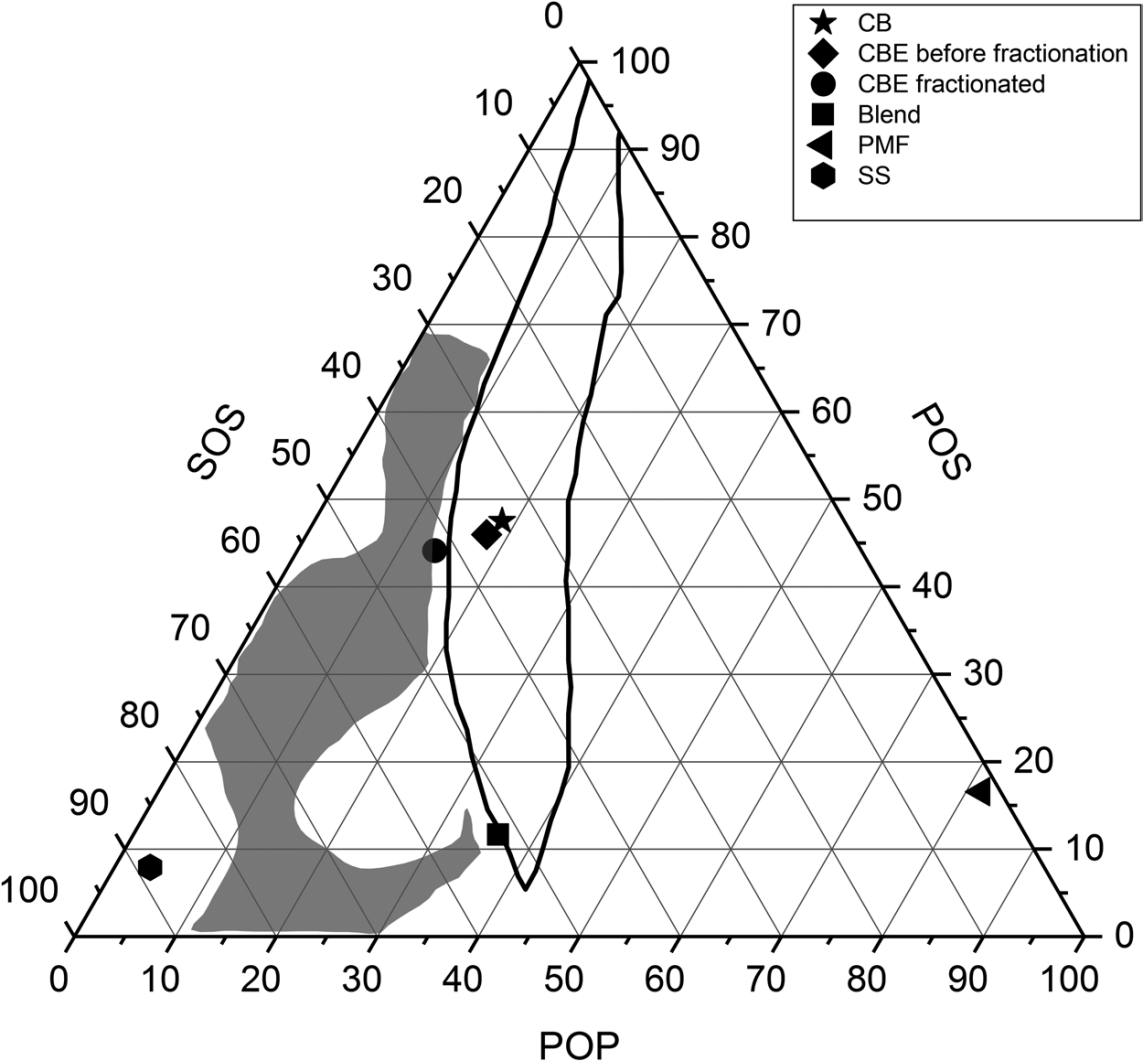
Ternary phase diagram of CB, CBE before and after fractionation, SS, PMF and SS/PMF (60:40 w/w).

In binary mixtures of POP/POS and POP/SOS, eutectic behaviour has been observed, while POS/SOS mixtures display ideal solid solution behaviour^72–74^. A ternary diagram of POP, POS, and SOS is shown in Fig. 3. PMF and SS are located at the base of the graph close to the POP and SOS legs. CB is located in the middle of the diagram. Padley^75^ defined a compositional zone area inside the ternary graph and stated that fats in this area should have the same tempering behavior as CB.

In a recent work, De Clercq *et al*.^69^ studied the physicochemical properties of eight commercial CBEs plus PMF and illipe butter and compared them with CB. They showed that all CBEs had comparable POS contents (12–13.8%), which was considerably lower than the amount found in CB. They showed in a POP/POS/SOS ternary graph that only one CBE was close to the stability boundary. In addition, they showed that these CBEs had different physical properties and tempering behaviors compared to CB. Ali *et al*.^76^ studied the melting behaviour and SFC of blended PMF, illipe butter, and sal stearin (PMF/IP/SLs) mixtures using a ternary phase diagram. They reported that since the amount of POS was lower in all blend ratios, none of them had physical properties similar to CB. They found eutectic interactions between all binary mixtures except IP/SLs and the addition of a small amount of PMF cause a drastic reduction in the melting point and SFC of binary blends. Also, addition of high amounts of PMF has a softening effect on CBE^77^. Koyano *et al*.^78^ showed eutectic behaviour between POP/SOS and POP/POS in a POP/POS/SOS ternary phase diagram. By increasing the concentration of POS and SOS, the melting point of the mixture increased, while addition of POP caused a reduction in the melting point. They mentioned that optimizing POP/SOS to formulate a proper CBE was critical and that the amount of POP should be as low as possible. In 2012, Bootello *et al*.^5^ showed that the phase behaviour of PMF and SS mixtures (containing 68.5% SOS) exhibited monotectic solution behavior, with partial solid solution behaviour at different ratios, but when the PMF content was increased to above 50% a strong softening effect was observed at lower temperatures.

### Melting point and enthalpy of fusion

**Figure 4:**
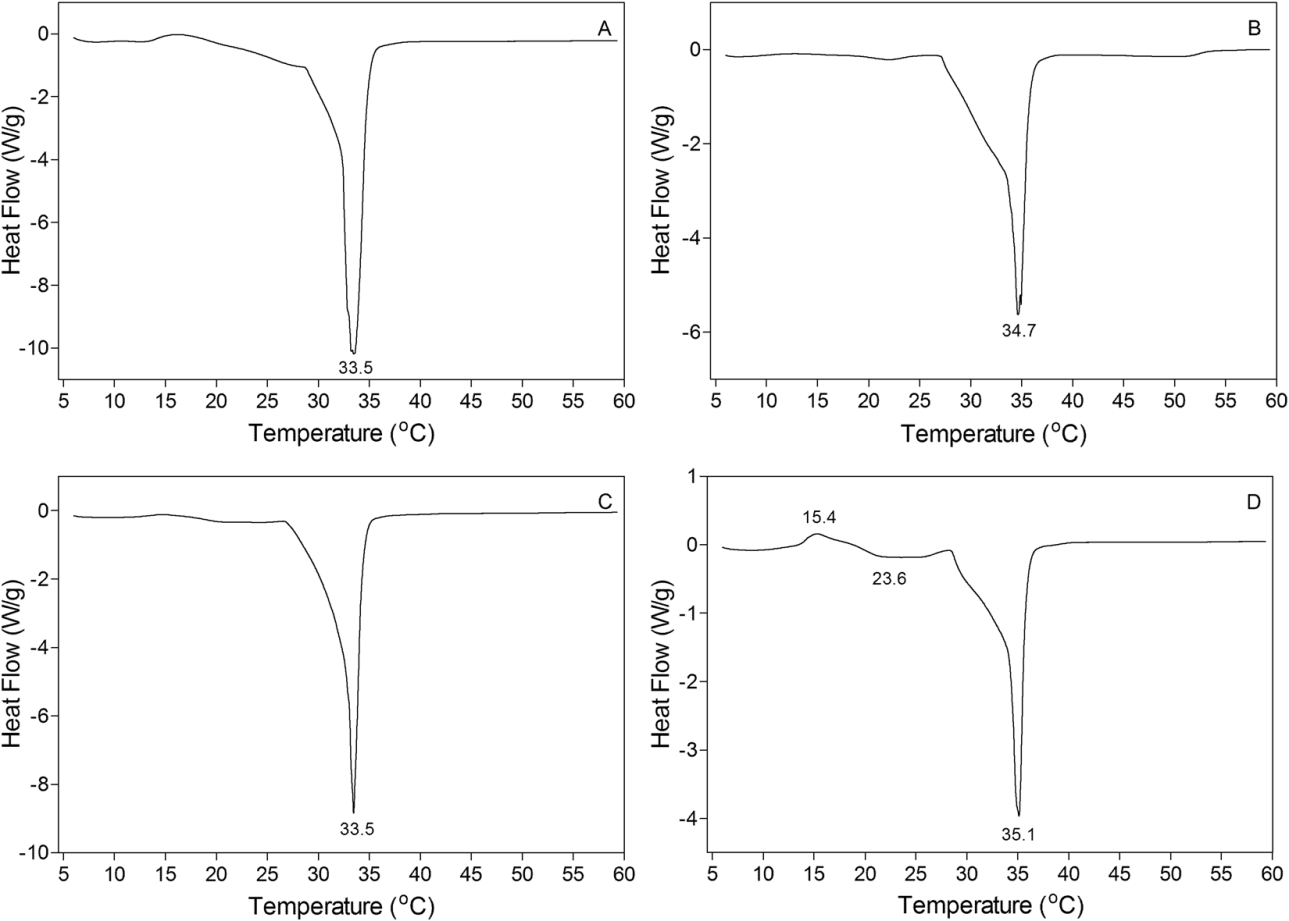
Differential scanning calorimetric heating thermograms of CB (**A**), CBE (**B**), CB/CBE (85:15 w/w) (**C**) and SS/PMF (60:40 w/w).

Katritzky *et al*.^79^ showed that melting point mainly is controlled by intermolecular forces, molecular symmetry, and molecular conformational degrees of freedom. Cocoa butters are usually obtained from cacao trees grown in various regions in the world with different climates and growth conditions that could affect their TAG composition and hence melting behaviour^55^. With such variability, CB can display melting points mainly between 32 °C to 34 °C in its β_V_ (form V) crystal form^80^. Many factors affect the melting behaviour and crystallization rate of CB, including processing conditions (refining, shearing and tempering), presence of minor components (phosphatides, glycolipids, free fatty acids, mono- and di-acylglycerols) and more importantly CB TAG profile and polymorphic form^71^. The melting points and enthalpies of CB, CBE, SS/PMF (60:40 w/w) and CB/CBE (85:15 w/w) blends are shown in Table 4 and Fig. 4. No significant difference between melting points of tempered CB, CBE and blend of CB/CBE (85:15 w/w) was detected (*P* < 0.05) (Table 4). In our study, tempered CB in the β_V_ form had a melting point of 33.6 °C that was very close to 33.8 °C for the melting point of CB in β_V_ polymorph reported by Wille and Lutton^81^. The melting point of tempered SS/PMF (60:40 w/w) was 35.1 °C, which was significantly higher than the melting point of CB, CBE, and their blend (CB/CBE (85:15 w/w)). This could be as a result of higher SOS contents (44.5%) in SS/PMF (60:40 w/w) mixture compared to the CB (27.7%) and CBE (34.8%). Moreover, the enthalpy of fusion for SS/PMF (60:40 w/w) was significantly lower than CB, CBE and their 85:15 (w/w) blend (*P* < 0.05). Padley^55^ showed that increasing SOS content in CB caused an increase in melting point and crystallization rate of CB at specific temperatures. The melting profile of SS/PMF (60:40 w/w) (Fig. 4D) showed a re-crystallization exothermic peak at 15.4 °C and a broad melting peak at 23.6 °C and a main endothermic peak at 35.1 °C.

**Table 4:** Melting points and enthalpies of fusion for CB, CBE, SS/PMF (60:40 w/w) and CB/CBE (85:15 w/w).

	CB	CBE	CB/CBE (85:15 w/w)	SS/PMF (60:40 w/w)
Melting point (°C)	33.6 ± 0.2^a^	34.4 ± 0.4^a,b^	33.5 ± 0.1^a^	35.1 ± 0.1^b^
Enthalpy (J/g)	148.0 ± 1.1^a^	139.8 ± 5.3^a,b^	158.0 ± 0.6^a,c^	103.3 ± 6.2^d^

Different superscript letters within each row indicate significant differences (*P* < 0.05) among samples.

In order to analyze melting profiles of tempered samples, first samples were cooled down from 23–24 °C to 5 °C followed with heating to 60 °C with the heating rate of 5 °C. The presence of about 6% low melting TAGs (mainly POO and SOO) in SS/PMF (60:40 w/w) and melt-mediated re-crystallization of POO and SOO in the blend during exothermic heating, could be reason of small melting and crystallization peaks in SS/PMF (60:40 w/w) thermogram. deMan *et al*.^82^, showed that the β’ polymorph of SOO has a melting point of 8.8 °C while, melting point of β crystal form of SOO was 23.7 °C. These results are very close to the melting point obtained for SOO in the most stable crystal form in this study (23.6 °C). Very similar results for melting profile of SOO were reported by Lutton^83^ and Daubert and Clarke^84^. On heating samples from 5 °C, SOO β’ was melted at 8–10 °C and subsequently recrystallized in the most stable form at 15.4 °C and finally β form was melted at 23.6 °C. Zhang *et al*.^85^ showed DSC heating of binary blends of immiscible metastable forms of SOO/SOS, SOO in crystal form β’_2_ was melted and re-crystallized to β’_1_ and further heating caused melting SOO β’_1_ at 27 °C. POO in the most stable crystal form has a melting point of 19.5 °C^83,86^ that is lower than storage temperature of samples after tempering (23–24 °C), so POO was in liquid form. Moreover, the undercooling temperature for POO crystallization in the α polymorph is much lower (−13.2 °C) than the starting temperature of heating (5 °C) in our DSC melting analysis^84^. Thus, we believe the exothermic and endothermic peaks before the main DSC melting peak for SS/PMF (60:40 w/w) are related to SOO than POO. However, further studies are required to clarify TAG molecular interactions in the temperature range of 5 °C to 25 °C.

### Solid Fat Content

**Figure 5:**
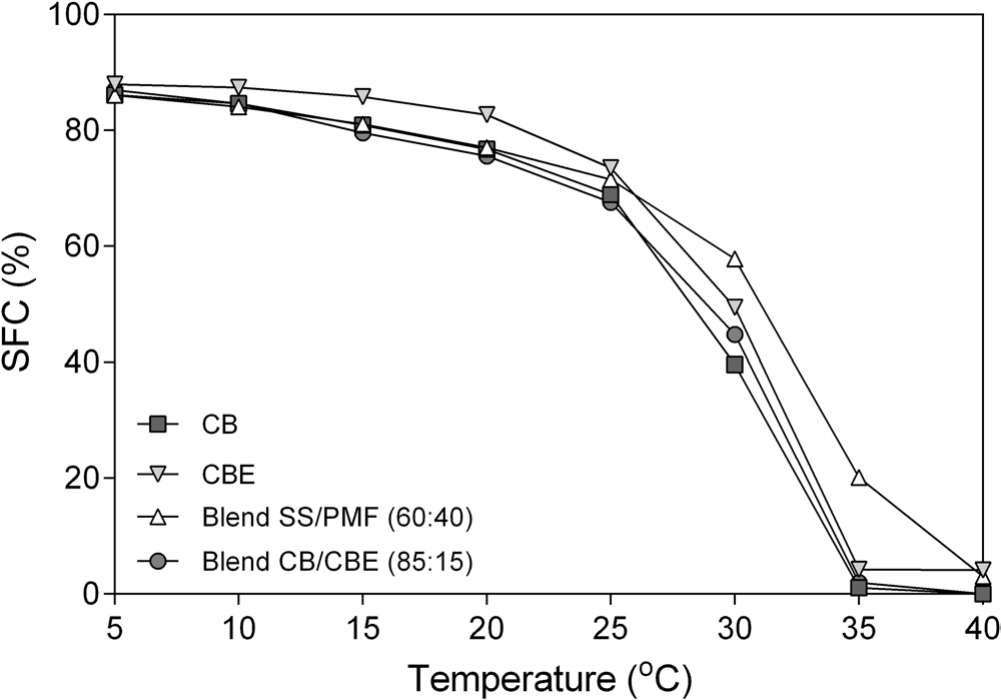
Solid fat content of CB, CBE, SS/PMF (60:40 w/w) mixture and CB/CBE (85:15 w/w) as function of temperature.

The solid fat content (SFC) of a fat is one of the most important physical properties of these materials, which can be used as an indicator of final product hardness, heat resistance and sensory characteristics, particularly for chocolate^87^. Similar to melting point, the SFC of CBs obtained from different origins can vary widely. For example, while tempered Brazilian CB had SFC of 66% at 20 °C, this amount for Malaysian CB at that temperature was 81%^55^. The SFC of a fat at specific temperature indicates the amount of solid crystalline TAGs that entrap liquid TAGs in their fat crystal network. The SFC is an important physical property of fats, such as CB, that can predict hardness. Moreover, the amount of solid fat and the steepness of SFC-temperature profiles can be correlated to sensory properties such as melting profile or waxiness in chocolate. The main factors that govern an SFC-temperature profile are the TAG profile and crystal polymorphic form^88^. The SFC of CB, CBE, SS/PMF (60:40 w/w) and CB/CBE (85:15 w/w) as function of temperature is shown in Fig. 5. The trend of SFC profile for all samples (CB, CBE, SS/PMF (60:40 w/w) and CB/CBE (85:15 w/w) was quite similar. Since the total amount of di-unsaturated TAGs (SOO and POO) in CBE was the lowest amount (2.5%) compared to CB (5.5%) and SS/PMF (60:40 w/w) blend (6.2%), CBE had the highest SFC at lower temperatures (5, 10, 15, 20 and 25 °C) compared to CB and SS/PMF (60:40 w/w) blend. Torbica *et al*.^87^ correlated the SFC value in specific temperature ranges to functional characteristics in confectionary products. For example, CB hardness was judged based on the SFC below 25 °C, heat resistance property by the SFC between 25 °C to 30 °C, and waxiness from SFCs above 35 °C. Marty-Terrade and Marangoni^89^ found that higher levels of POO and SOO in Brazilian CB. The largest decline relative to CB and SS/PMF blend in SFC as a function of temperature was obtained for the CBE from 25 to 35 °C. The steep drop in SFC from 25 °C to 35 °C can be correlated to a rapid melting of CB in the mouth, which is also an indication of flavour release at body temperature^90^. The SFC-temperature profile of the CBE mimicked that of CB. In contrast, a more gradual decrease (less sharp) in SFC as a function of temperature was obtained for SS/PMF (60:40 w/w). The CBE and SS/PMF (60:40 w/w) blend were not completely melted at 40 °C and had an SFC of 4.1% and 3.0%, respectively. A higher SFC at 40 °C could be due to the presence of tri-saturated TAGs (PPP, PPS, PSS and SSS) in the CBE and SS/PMF (60:40 w/w) blend, which were not completely melted at 40 °C^90^. Moreover, comparing the TAG profile of CBE vs SS/PMF (60:40 w/w) (Table 3), showed that solvent fractionated CBE still had 3.8% StStSt, while CB and SS/PMF (60:40 w/w) contained 1.8% and 2.9%, respectively. TAG analysis of the CBE before and after fractionation showed the most of the SSS and PSS had been eliminated after fractionation, while no significant change in the amount of PPP was detected.

To sum up, the high amount of StStSt and DAGs in CBE and SS/PMF (60:40 w/w) could be a reason for the higher SFC at 40 °C compared to CB. Similar results were reported by Depoortere^91^, who showed that the SFCs of some tempered commercial CBEs containing about 2% tri-saturated TAGs were in the range of 2–4% at 40 °C. Timms and Stewart^92^ showed that increasing SSS content in CB from 1.4% to 3.6% caused ad increase in SFC from 1.3% to 5.2% at 35 °C. Kadivar *et al*.^27^ synthesized a CBE using enzymatic acidolysis between high stearic high oleic sunflower oil (HSHO) or high oleic sunflower oil (HOSO), plus a mixture of palmitic and stearic acids. The SFC of 25% and 50% (w/w) blend of HOSO CBE and HSHO CBE mixed with CB had significantly lower SFC between 20–30 °C in comparison with CB. The CBE produced using HSHO had an SFC of 3.1% at 40 °C. In our study, on the other hand, the SFCs for neat CBE and CB/CBE (85:15 w/w) was higher than that of CB (*P* < 0.05), possibly suggesting increased heat resistance and hardness^69^. Although the SFC of neat CBE was significantly higher than CB at 35 and 40 °C, no significant difference in SFC between CB:CBE 85:15% and CB was detected (*P* < 0.05). Klagge and Gupta^90^ showed that the addition of 15% commercial CBE to Cameroon CB, increased the SFC. The SFC for their CB:CBE (85:15 w/w) blend at 10 °C, 20 °C, 25 °C, 30 °C and 35 °C was 82%, 76.4%, 68.6%, 42.2% and 0.7%, respectively. The SFC of the CB:CBE (85:15 w/w) blend in our study at 10 °C, 20 °C, 25 °C, 30 °C and 35 °C was 84.6%, 75.6%, 67.6%, 44.8% and 2%, respectively.

### Crystal structure and polymorphism

The reason for the multiple melting profiles of TAGs was poorly understood until the work of Clarkson and Malkin in 1934^93^. They used X-ray analysis and calorimetry to demonstrate that the different melting points for the same TAG were associated with different crystal polymorphic forms. For the first time, a nomenclature for the polymorphic forms of TAGs was proposed. Ten years later in 1945, Lutton^94^ studied the crystal structures and polymorphism of tristearin and some of its homologs (trilaurin, trimyristin and tripalmitin). They corrected the nomenclature for tristearin polymoprhs from Malkin’s γ, α, and β, in order of increasing melting point, to α, β’ and β crystal forms. In the same year, extensive work of Filer *et al*.^95^ on X-ray diffraction and calorimetry of purified synthetic tri-acid TAGs crystallized from solvent at different rates was published. They found a high correlation between TAG molecular weights and melting points and long-spacings (reflections originating from (001) planes). In 1966, a further refinement of the nomenclature of polymorphs, including sub-α and different β crystal forms (β_1_ and β_2_) was introduced by Larsson^96^.

**Figure 6:**
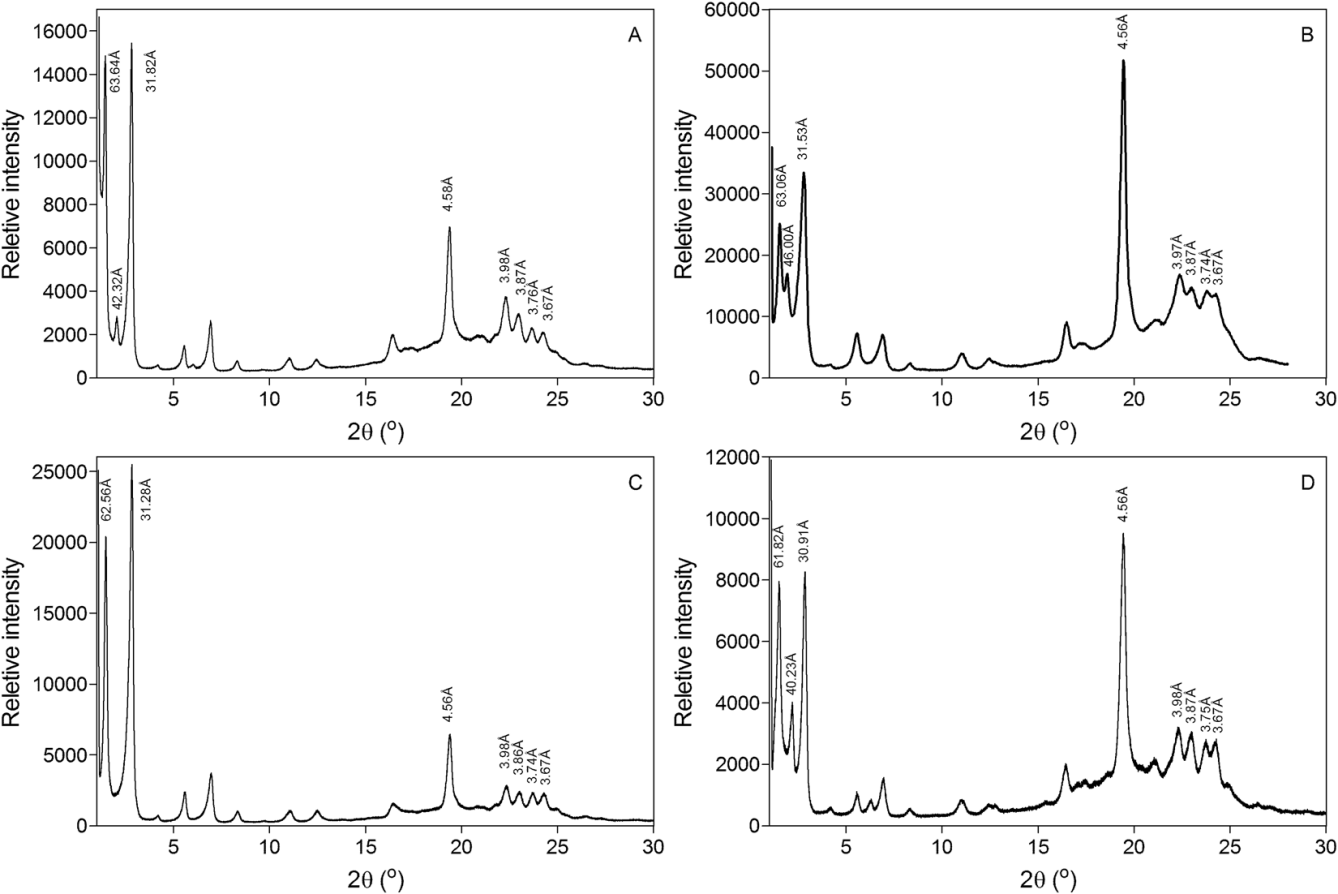
Small and wide-angle powder X-ray diffraction spectra of CB (**A**), CBE (**B**), CB/CBE (85/15 w/w) (**C**) and SS/PMF (60:40 w/w).

By the late 1920’s, only three polymorphs of CB were known, with melting points of 24 °C, 29 °C and 34 °C^97^. Many years later in 1960, an extensive study was conducted on CB and bloom formation by Vaeck^98^ using microscopic analysis and melting point determination. Based on their results, four crystal forms were proposed for CB, γ, α, β’ and β in order of increasing melting point of 17 °C, 23 °C, 28 °C and 35 °C, respectively. The fifth polymorphic form was introduced by Duck^99^ with a melting point of 33 °C. In 1966, the most comprehensive report on CB polymorphic forms was published by Wille and Lutton^81^. They identified six crystal polymorphic forms for CB with different melting points and dilatometric data. The melting points of six polymorphic forms were; form I (17.3 °C), form II (23.2 °C), form III (25.5 °C), form IV (27.5 °C), form V (33.8 °C) and form VI (36.3 °C). Since CB polymorphic forms have great influence on the physical and sensory characteristics of chocolate such as melting point, SFC, hardness, gloss and snap, obtaining the proper crystal structure and polymorphic form for our synthesized CBE was a major priority in this study. Tempered cocoa butter in the form V (β_2_) crystal form provides the desired physical and sensory characteristics in chocolate, such as gloss, snap, hardness, melting profile and flavor release. Small and wide angle powder X-ray diffraction spectra of tempered CB, CBE, SS/PMF (60:40 w/w) and CB/CBE (85:15 w/w) is shown in Fig. 6. Tempered CB in our study showed diffraction peaks in the wide-angle x-ray scattering (WAXS) region at 4.58 Å (vs), 3.98 Å (s), 3.87 Å (m), 3.76 Å (m) and 3.67 Å (m) (Fig. 1A). In this field, vs, s, m, w represented “very strong”, “strong”, “medium” and “weak” peak intensities. Wille and Lutton (1966)^81^ reported a quite similar diffraction pattern for tempered CB in form V at 4.58 Å (vs), 3.98 Å (s), 3.87 Å (m), 3.75 Å (m), and 3.67 Å (w). Tempered CBE and SS/PMF (60:40 w/w) blend also displayed very similar diffraction patterns. The only major difference was observed for SS/PMF (60:40 w/w) blend that showed an equal intensity for diffraction peaks at 3.98 Å and 3.87 Å while for the other samples the intensity of the peak at 3.98 Å was stronger than that at 3.87 Å (Fig. 1B and D). The d-spacing corresponding to lamellar sizes in the small angle x-ray scattering (SAXS) region of all samples showed a 3-L crystal structure form with the d_001_ = 63.4 Å for form CB, d_001_ = 63.1 Å for CBE, d_001_ = 62.6 Å for CB:CBE (85:15 w/w) and d_001_ = 61.8 Å for SS:PMF (6:4 w/w) blend (Fig. 6A–D). Although in the SAXS region, a 2 L crystal structure was obtained at d_001_ = 42.3 Å for CB, d_001_ = 46.0 Å for CBE and 40.2 for SS/PMF (60:40 w/w) blend. Where “2 L” or “3 L” refer to the number of fatty acid chain lengths within the long axis of the TAG unit cell. Loisel *et al*.^100^ showed a similar peak in the SAXS region (d_001_ = 44.4 Å) for tempered CB in crystal form V. X-ray diffraction as a function of temperature (XRDT) analysis (simultaneous DSC and XRD analysis) of CB in β_V_ polymorph showed this peak in the SAXS region that disappeared after heating CB at 37.5 °C. They concluded that the peak at 44.4 Å (2 L crystal packing) corresponded to a β crystal form of tri-saturated TAGs in CB. Davis and Dimick^101^ showed the TAG analysis of a high melting fraction which fractionated out after six hours static incubation of melted CB at 26.5 °C. This high melting fraction contained high amounts of PPS, PSS and SSS. In our study, the total amount of tri-saturated TAGs (PPP, PPS, PSS, and SSS) in CB, CBE and SS/PMF (60:40 w/w) blend was 1.8%, 3.8% and 2.9%, respectively (Table 3).

### Crystal morphology of CB, CBE, CB/CBE (85:15 w/w) and SS/PMF (60:40 w/w) blends

**Figure 7:**
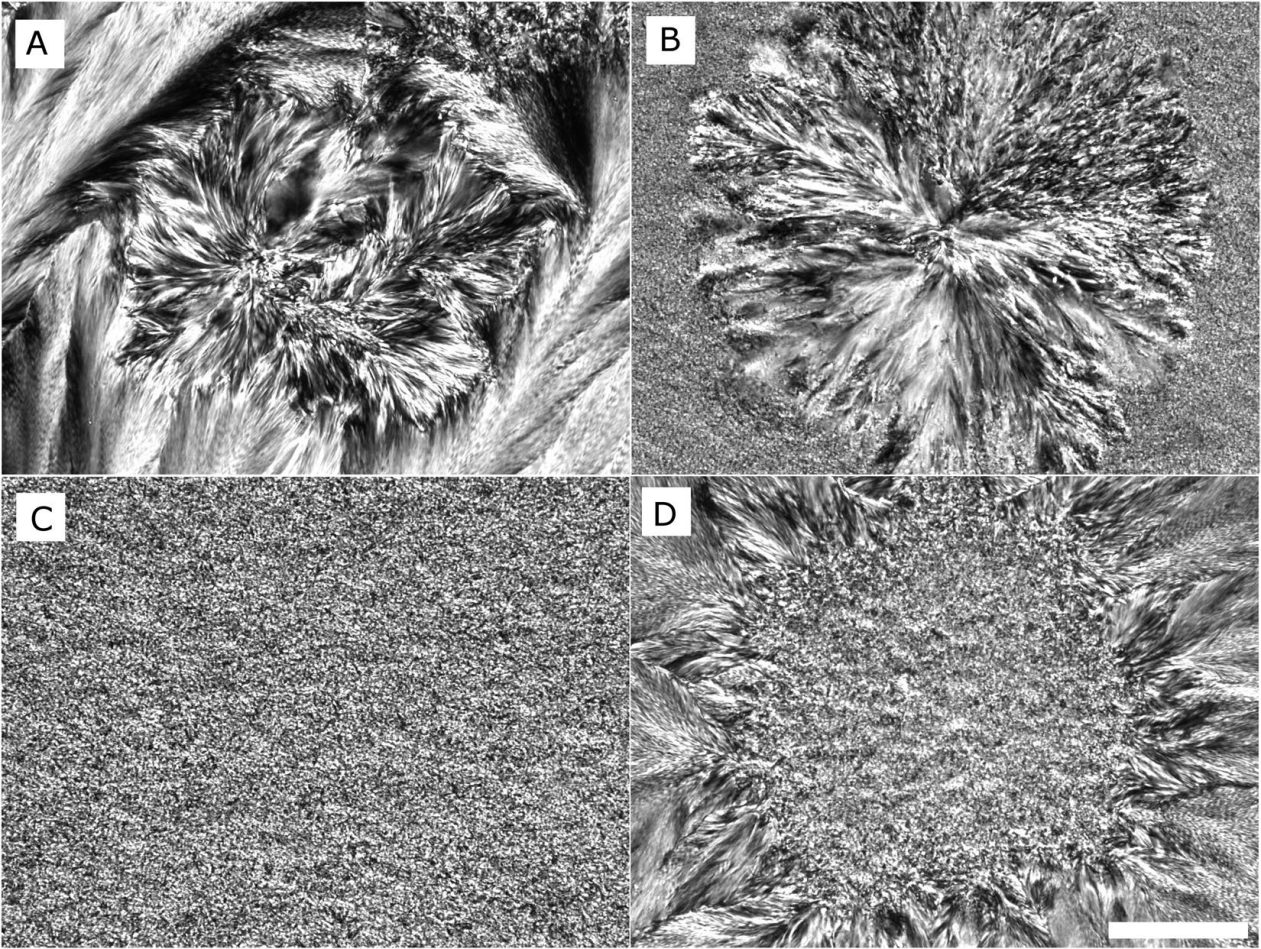
Polarized light micrographs of CB (**A**), CBE (**B**), CB/CBE (85:15 w/w) (**C**) and SS/PMF (60:40 w/w) after one-week storage at 22–23 °C. The bar represents 20 µm.

The morphology (shape and size) of CB crystal forms and their interaction to make clusters and a network plays an important role in the physical, rheological and sensory characteristics of chocolate^102^. Many factors such as TAG composition, crystallization temperature and time, minor impurities (free fatty acids, phosphatides and glycolipids), crystal polymorphism and processing conditions have effect on fat crystal microstructure formation^89,103^. Polarized light microscopy (PLM) allows the observation of individual and aggregated crystal clusters in CB. A comprehensive study on morphology of CB crystals in different crystal polymorphic forms was reported by Vaeck^98^. PLM images of CB, CBE, CB/CBE (85/15 w/w) and SS/PMF (60:40 w/w) blends, after one week static crystallization at 22 °C are shown in Fig. 7. A mixture of spherical and feather-like crystallites was observed for CB, CBE and SS/PMF (60:40 w/w) blend (Fig. 7A,B and D). While, a continuous dense uniform phase with a lacy network of very small crystals was observed for CB blended with CBE (15/85%) (Fig. 7C). After 7 days incubation at 22 °C, spherulites (40–60 µm) were clustered forming a feather-like crystal network (Fig. 7A), while larger spherulitic microstructures (100–500 µm) were observed for SS/PMF (6:4 w/w) blend (Fig. 7D). The difference in microcrystalline structure between CB/CBE (85:15 w/w) and CB, CBE and SS/PMF (60:40 w/w) blend could be due to phase separation, or TAG fractionation, that did not occur in the CB/CBE (85/15 w/w) mixture. Similar results were obtained by Kadivar *et al*.^27^, they showed addition of HOSO and HSHO CBEs to CB hampered crystal formation (dense granular structure with no spherulitic crystals) in the blends. They commented that the presence of DAGs and SOO in CBEs inhibited crystal growth. The morphology of CB micro-crystals formed through static crystallization is mainly a function of temperature^103,104^. McGauley and Marangoni^103^ showed after one-day incubation of CB at 22 °C, clustered spherulites in the β’ crystal form were observed. They showed that a 4–5 week incubation at 22 °C was necessary to obtain CB microcrystalline structures in the β crystal polymorph. However, classifying fat crystal polymorphic form based on the microstructure and morphology of crystals is challenging because similar crystal morphologies can be obtained for β’ or β polymorphic forms^105^. However, based on the small crystals size in CB/CBE (85:15 w/w) mixture, it appeared that they were in β’ crystal forms while other samples (CB, CBE and SS/PMF (60:40 w/w) with large clustered crystals could be in a mixture of both β’ and β or in an intermediate crystal polymorphic forms^106^. The difference in morphology of crystals in CB/CBE (85:15 w/w) could be the result of the tri-saturated TAGs content in this sample, which could had an effect on nucleation and growth rates of the crystallizing fat. The amount of tri-saturated TAGs (PPP, PPS, PSS and SSS) in CB/CBE (85:15 w/w) blend was the lowest (1.4%) compared to CB, CBE and SS/PMF (60:40 w/w) blend (1.8%, 3.8% and 2.9%, respectively). Moreover, high-melting tri-saturated TAG crystals which were formed during the initial steps of crystallization in CB, CBE and SS/PMF (60:40 w/w) blend could provide a distinct crystal morphology with individual spherical structures that were observed in all samples except in CB/CBE (85:15 w/w) blend^101^.

## Conclusion

In this study, a CBE was synthesized from a 60:40 (w/w) mixture of a commercial enzymatically synthesized shea stearin and palm mid fraction, catalysed by 10% (w/w) immobilized Lipozyme RM IM. The EIE reaction was optimized after pre-conditioning steps (removing air and restricting moisture content in reaction medium). The EIE reaction was conducted at 65 °C for four hours. This protocol was optimal to synthesize a CBE with a fatty acid and TAG composition very similar to that of CB. Moreover, physical properties of synthesized CBE were also very similar to those of CB. It was also shown that fractionation can be used to remove undesirable TAGs (di-unsaturated and tri-saturated). Overall, we have shown here that it is possible to synthesize a CBE with molecular structure and physical properties that resemble cocoa butter very closely. This was achieved in a two step solvent-free reaction, with minimal downstream processing. We believe that this strategy is optimal for the industrial scale production of a true chocolate fat mimetic^31^.

## Data Availability

All data will be provided in Excel files to anyone requesting it.
